# Transition-state destabilization reveals how human DNA polymerase β proceeds across the chemically unstable lesion N7-methylguanine

**DOI:** 10.1093/nar/gku554

**Published:** 2014-06-25

**Authors:** Myong-Chul Koag, Yi Kou, Hala Ouzon-Shubeita, Seongmin Lee

**Affiliations:** Division of Medicinal Chemistry, College of Pharmacy, The University of Texas at Austin, Austin, TX 78712, USA

## Abstract

N7-Methyl-2′-deoxyguanosine (m7dG) is the predominant lesion formed by methylating agents. A systematic investigation on the effect of m7dG on DNA replication has been difficult due to the chemical instability of m7dG. To gain insights into the m7dG effect, we employed a 2′-fluorine-mediated transition-state destabilzation strategy. Specifically, we determined kinetic parameters for dCTP insertion opposite a chemically stable m7dG analogue, 2′-fluoro-m7dG (Fm7dG), by human DNA polymerase β (polβ) and solved three X-ray structures of polβ in complex with the templating Fm7dG paired with incoming dCTP or dTTP analogues. The kinetic studies reveal that the templating Fm7dG slows polβ catalysis ∼300-fold, suggesting that m7dG in genomic DNA may impede replication by some DNA polymerases. The structural analysis reveals that Fm7dG forms a canonical Watson–Crick base pair with dCTP, but metal ion coordination is suboptimal for catalysis in the polβ-Fm7dG:dCTP complex, which partially explains the slow insertion of dCTP opposite Fm7dG by polβ. In addition, the polβ-Fm7dG:dTTP structure shows open protein conformations and staggered base pair conformations, indicating that N7-methylation of dG does not promote a promutagenic replication. Overall, the first systematic studies on the effect of m7dG on DNA replication reveal that polβ catalysis across m7dG is slow, yet highly accurate.

## INTRODUCTION

Chemical modification of DNA by endogenous and exogenous methylating agents such as *S*-adenosylmethionine and *N*-methyl-*N*-nitrosourea generates a wide array of genotoxic lesions, with N7-methyl-2′-deoxyguanosine (m7dG) comprising ∼70–80% of the total methylated lesions (1–3). An enzymatically-introduced m7dG adduct has been used to determine specific protein–DNA interactions (4) and characterize thermodynamic properties of m7dG (5). In addition, a nonenzymatically-introduced m7dG adduct has been extensively used in DNA sequencing methods (6). The formal positive charge at N7 of m7dG weakens the glycosidic bond and facilitates spontaneous depurination, which produces abasic sites that can give rise to G to T transversion mutations (7). In basic conditions, m7dG can undergo imidazole ring opening to generate mutagenic 5-*N*-methyl-2,6-diamino-4-hydroxyformamidopyrimidine (Methyl-FAPy) (8). Most organisms prevent the promutagenic spontaneous depurination using alkylation-damage repair DNA glycosylases, which excise N7-methylguanine from m7dG to generate abasic sites and handover the cytotoxic intermediates to AP endonuclease, a downstream enzyme in base excision DNA repair pathway (9).

**Figure 1.**
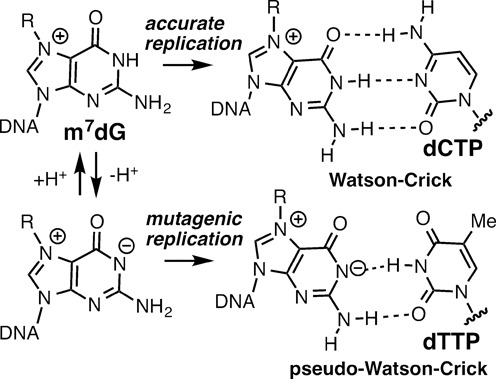
Structures of Watson–Crick m7dG:dCTP and pseudo-Watson–Crick m7dG:dTTP base pairs.

Despite the predominance of m7dG in nonenzymatic methylation, the effect of intact m7dG (i.e. not depurinated) on DNA replication has remained elusive, even after several decades since the first observation of m7dG lesion (3). Although m7dG undergoes spontaneous depurination much faster than dG (5), the lesion in duplex DNA is quite stable (*t*_1/2_ of 6 days) (10) and persistent in genomic DNA at the level of several adducts per 10 million bases (11). In addition, the N7-methylation of dG decreases the p*K*_a_ of N1 of m7dG to a value of ∼7 (12,13), and might facilitate the formation of pseudo-Watson–Crick m7dG:dTTP base pair during DNA replication by forming zwitterionic form of m7dG (Figure 1) (12,14), which could promote G to A transition mutations. The effects of abasic sites on biological processes have been extensively investigated (15), but there have been only a few reports that describe the effects of intact m7dG on biological processes. The presence of m7dG in methylated CpG sequence has shown to preclude binding of methyl-CpG-binding domain 1 (16), and m7dG in DNA inhibits binding of major-groove-interacting proteins (4). A systematic study on the effect of m7dG on DNA replication has not been reported so far. Such study would be of significance because it may provide insights into the effect of the predominant N7-dG alkylation adducts on DNA replication and mutagenesis.

**Figure 2.**
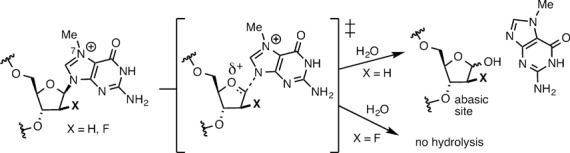
Inhibition of depurination of m7dG via 2′-F-mediated transition-state destabilization

A systematic investigation of the effect of m7dG on DNA replication has been hampered due in part to the lack of an efficient method that generates sufficient quantities of site-specifically incorporated m7dG-containing DNA for kinetic and structural studies (17,18). To investigate such effect, we employed a 2′-fluorine-mediated transition-state destabilization strategy (Figure 2), which was recently used to site-selectively introduce 2′-fluoro-m7dG (Fm7dG), a stable nonhydrolyzable m7dG analogue, into DNA and to solve the crystal structure of Fm7dG-containing DNA in complex with AlkA (19).

As an initial effort to elucidate the effect of m7dG on DNA replication and mutagenesis, we chose human DNA polymerase β (polβ) as a model system. The X-family DNA polymerase polβ is an error-prone DNA polymerase lacking an intrinsic proofreading 3′ to 5′ exonuclease activity. Polβ participates in base-excision DNA repair by filling a short nucleotide gap generated by the action of DNA glycosylases and AP endonuclease. In addition to its role in base-excision DNA repair, polβ has been implicated in the translesion synthesis of various types of DNA damage such as 8-oxoguanine, cisplatin-GG intrastrand cross-link adducts and UV-induced cyclobutane pyrimidine dimers (20). The plethora of kinetic and structural data available for polβ with correct and incorrect insertion facilitate interpretation of new structures (21,22), and the small size (39 kDa) of polβ makes it as an ideal model DNA polymerase for the studies of nucleotidyl transfer chemistry and substrate discrimination mechanisms.

To evaluate the effect of m7dG on the polymerase activity of polβ, we determined kinetic parameters for the incorporation of dCTP opposite the chemically stable Fm7dG by polβ and solved three ternary structures of polβ in complex with the templating Fm7dG paired with incoming nonhydrolyzable 2'-deoxycytidine-5'-triphosphate (dCTP) or 2'-deoxythymidine-5'-triphosphate (dTTP) analogues. Herein, we report the first kinetic studies that show the effect of Fm7dG on polymerase activity. We also present a crystal structure of polβ inserting dCTP opposite the templating Fm7dG, which reveals for the first time the base-pairing nature of Fm7dG:dCTP in the confines of a DNA polymerase. Our studies provide insights into the effect of N7-alkyl-dG lesion on DNA replication and mutagenesis.

## MATERIALS AND METHODS

### Synthesis of the Fm7dG phosphoramidite

The Fm7dG phosphoramidite was prepared starting from a ribose derivative according to the synthetic procedures described previously (19).

### DNA sequences used for kinetic and X-ray crystallographic studies

The oligonucleotides for kinetic assays (upstream primer, 5′-FAM/GGGGGCTCGTAAGGATTC-3′, downstream primer, 5′-phosphate/AGTCGG-3′ and template, 5′-CCGACT(X)GAATCCTTACGAGCCCCC-3′, X = dG, 2FdG or Fm7dG) were synthesized by Midland Certified Reagent Co. (Midland, TX, USA) and Integrated DNA Technologies (Coralville, IA, USA). The template DNA was prepared via solid-phase DNA synthesis using ultramild deprotection conditions (50 mM K_2_CO_3_ in MeOH, 25°C, 24 h). The DNA sequences used for co-crystallization were 5′-CCGAC(Fm7dG)TCGCATCAGC-3′ for template, 5′-GCTGATGCGA-3′ for the upstream primer and 5′-phosphate/GTCGG-3′ for the downstream primer. The oligonucleotides were annealed to give a single-nucleotide gapped DNA, which was used for the crystallization of both binary and ternary polβ complex structures (21).

### Expression and purification of human DNA polymerase β

Polβ used for kinetic and crystallographic studies was expressed and purified from *Escherichia coli* as described previously (22).

### Steady-state kinetics of nucleotide incorporation opposite Fm7dG by polβ

Steady-state kinetic parameters for dCTP insertion opposite templating dG, 2′-fluoro-2′-deoxyguanosine (FdG) and Fm7dG by polβ were determined using the conditions described previously (22). DNA substrates containing a single-nucleotide gap opposite the templating dG, FdG and Fm7dG were prepared by annealing the template oligonucleotide (5**′**-CCGACT(X)GAATCCTTACGAGCCCCC-3**′**; X = dG, FdG and Fm7dG) with the upstream (5**′**-FAM/GGGGGCTCGTAAGGATTC-3**′**) and the downstream primers (phosphate/AGTCGG-3**′**) at 95°C for 3 min followed by slow cooling to 4°C. Recessed DNA substrates that do not have the downstream primer were prepared similarly. Polβ activities were determined using the reaction mixture containing 50 mM Tris-HCl pH 7.5, 5 mM MgCl_2_, 100 mM KCl, 80 nM substrate DNA and varying concentration of the incoming nucleotide. The nucleotidyl transfer reactions were initiated by adding polβ and incubated at 37°C for 2 min, and quenched by adding a stop solution containing 95% formamide, 45 mM Tris-borate, 20 mM ethylenediaminetetraacetic acid , 0.1% bromophenol blue and 0.1% xylene cyanol. The nucleotidyl transfer reaction products were resolved on 18–20% denaturing urea polyacrylamide gels, and the product formation was analyzed using a PhosphorImager. The efficiency of nucleotide insertion was calculated as *k*_cat_/*K*_m_. The relative efficiency of dCTP incorporation opposite Fm7dG was determined as *f* = (*k*_cat_/*K*_m_)_[dCTP:Fm7dG]_/(*k*_cat_/*K*_m_)_[dCTP:dG]_.

### Co-crystallization and structure determination of polβ-DNA binary and ternary complexes

The binary and ternary polβ complexes with templating Fm7dG were crystallized using the similar conditions described previously (21). Diffraction data were collected at 100 K at the beamline 5.0.3 at the Advanced Light Source, Lawrence Berkeley National Laboratory. All diffraction data were processed using HKL2000. Structures were solved by molecular replacement using a gapped binary complex structure with an open conformation (PDB ID: 1BPX) and a ternary complex structure with a closed conformation (PDB ID: 1BPY) as the search models. The model was built using Coot and refined using Phenix.

## RESULTS

### Kinetic studies

To assess whether m7dG at the templating position affects the polymerase activity of polβ, we determined the kinetic parameters for the polβ-catalyzed insertion of dCTP and dTTP opposite the templating dG, FdG and Fm7dG (Table 1 and Supplementary Figure S1). In the absence of the 5′-phosphorylated downstream primer (5′-phosphate/AGTCGG-3′), the efficiency (*k*_cat_/*K*_m_) for dCTP insertion opposite the templating dG is very low. The use of the downstream primer dramatically increases the insertion efficiency (∼1100-fold), which is consistent with previous kinetic results that show a 300-fold increase in the catalytic efficiency for a 5′-phosphorylated single-nucleotide gapped DNA relative to a nongapped DNA (23). The efficiencies for dCTP insertion opposite the templating FdG and dG in the single-nucleotide gapped DNA are almost the same, indicating that 2′-fluorination has a minimal effect on polβ catalysis. In stark contrast, the presence of the templating Fm7dG increases *K*_m_ ∼10-fold and decreases *k*_cat_ ∼30-fold compared with dG, thereby reducing the relative efficiency for dCTP insertion opposite Fm7dG by ∼300-fold (Table 1). The sharp decrease in the relative efficiency is surprising, because the N7-methyl moiety perturbs neither the Watson–Crick H-bonding nor the minor-groove edges of dG. To evaluate whether the p*K*_a_ of Fm7dG contributes to the decrease in the relative efficiency, we determined kinetic parameters for dCTP insertion opposite Fm7dG at different pH conditions (pH 6.5 and 8.5). The relative efficiency increases only ∼2-fold when pH changes from 6.5 to 8.5, indicating that the p*K*_a_ of Fm7dG does not significantly contribute to the observed reduction in the relative efficiency. In addition to the Fm7dG:dCTP kinetics, we wanted to examine the effect of the N7-methylation on the formation of G:T mismatches, which comprise about 60% of polβ-induced spontaneous mutations (24), by determining kinetic parameters for dTTP insertion opposite Fm7dG. Surprisingly, the dTTP incorporation was not observed, suggesting that the templating m7dG greatly deters the dTTP insertion opposite the lesion in the active site of polβ.

**Table 1.** Kinetic parameters for insertion of dCTP and dTTP opposite templating dG, 2′-deoxy-2′-F-dG (FdG) and Fm7dG by polβ

Template:dNTP	pH	*K*_m_ (μM)	*k*_cat_ (10^−3^ s^−1^)	*k*_cat_/*K*_m_ (10^−3^ s^−1^/μM)	*f*^a^
dG:dCTP^b^	7.5	269.55 ± 18.87	7.56 ± 0.38	0.03	
dG:dCTP^c^	7.5	0.59 ± 0.03	20.38 ± 0.50	34.54	1
FdG:dCTP^c^	7.5	0.34 ± 0.07	12.52 ± 0.53	36.82	1.1
Fm7dG:dCTP^b,d^	7.5				
Fm7dG:dCTP^c^	6.5	3.86 ± 0.09	0.40 ± 0.06	0.10	2.9 × 10^−3^
Fm7dG:dCTP^c^	7.5	6.01 ± 0.75	0.69 ± 0.03	0.11	3.2 × 10^−3^
Fm7dG:dCTP^c^	8.5	3.87 ± 0.07	0.75 ± 0.02	0.19	5.5 × 10^−3^
Fm7dG:dTTP^c,d^	7.5				

^a^Relative efficiency: (*k*_cat_/*K*_m_)_[dCTP:dN]_/(*k*_cat_/*K*_m_)_[dCTP:dG]_.

^b^A recessed DNA (i.e. DNA without the downstream primer) was used as a substrate.

^c^Single-nucleotide gapped DNA was used as a substrate.

^d^Nucleotide incorporation was not observed.

### Binary structure of polβ in complex with DNA bearing a single-nucleotide gap opposite Fm7dG

**Figure 3.**
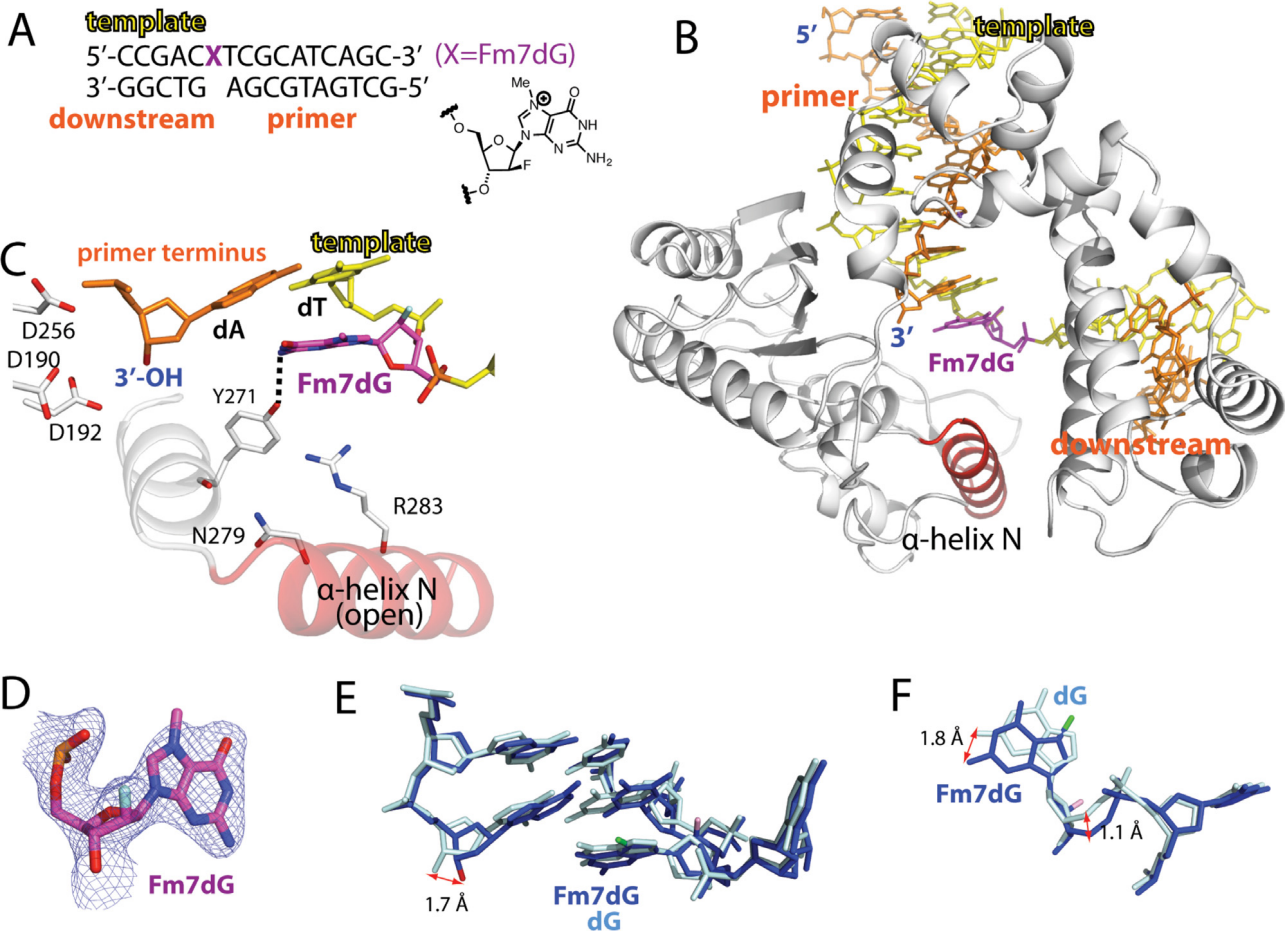
Single-nucleotide gapped binary structure of polβ in complex with DNA containing templating Fm7dG (PDB ID: 4O5C). (**A**) The DNA sequence used for crystallography and chemical structure of Fm7dG are shown. The downstream primer contains 5′-phosphate. (**B**) Overall crystal structure of the Fm7dG gapped binary complex. Polβ is shown in white. The α-helix N bearing the minor groove recognition amino acid residues Asn279 and Arg283 is shown in red. The template strand is shown in yellow and the upstream and the downstream primers are shown in orange. The templating Fm7dG is indicated. (**C**) Active-site structure. The α-helix N is in an open conformation. Y271 is H-bonded to N2 of Fm7dG (**D**) A 2*F*_o_-*F*_c_ electron density map is contoured at 1σ around Fm7dG. Templating Fm7dG adopts the 2′-endo sugar pucker and the *anti*-base conformation. (**E**) Comparison of the Fm7dG gapped structure (blue) with published dG gapped structure (pale cyan, PDB ID: 1BPX). Distance between the 3′-OHs of the primer terminus is indicated. (**F**) Comparison of templating Fm7dG (blue) with templating dG (pale cyan) in (E).

To evaluate whether m7dG at the templating position perturbs the conformation of DNA and protein, we solved a binary structure of polβ bound to DNA containing a single-nucleotide gap opposite the templating Fm7dG (Figure 3A; see Table 2 for refinement statistics). The gapped binary structure, refined to 2.4 Å resolution, indicates that whereas Fm7dG does not induce any substantial distortion of the polβ conformation, it induces a slight conformational change of DNA near the lesion (Figure 3B). The overall structure of the Fm7dG gapped binary complex is similar to that of a published dG gapped structure (PDB ID: 1BPX, RMSD = 0.619 Å, Figure 3E) (21). Polβ assumes an open protein conformation. The α-helix N bearing the minor-groove recognition amino acids, Asn279 and Arg283, does not engage in H-bonding interactions with DNA. The templating Fm7dG adopts an *anti*-base conformation and C2′-endo sugar pucker, as does templating dG (Figure 3C and D). Although the 2′-F is evident on the electron density map, the N7-methyl moiety is not. Fm7dG at the templating position induces a minor conformational change at the 5′ and the 3′ sides of the m7dG lesion (Figure 3E). Interestingly, the 3′-OH of the primer terminus moves 1.7 Å toward the templating base relative to the position observed in published gapped structure (Figure 3E). The base moiety of templating Fm7dG slightly rotates toward the minor groove and the 5′ side of Fm7dG shifts ∼1 Å from the position observed in the templating dG-containing structure (Figure 3F). Overall, the Fm7dG gapped binary complex structure indicates that templating Fm7dG induces a slight conformational change of DNA, especially around the Fm7dG lesion.

### Ternary structure of polβ incorporating dCTP analog opposite templating Fm7dG

To gain insight into the observed slow insertion of dCTP opposite Fm7dG by the enzyme and to elucidate the base-pairing properties of Fm7dG:dCTP in the confines of the enzyme active site, we solved a ternary structure of polβ with templating Fm7dG base-paired with the incoming nonhydrolyzable dCTP analog dCMPNPP (dCTP* hereafter). The nonhydrolyzable dCTP analog contains a bridging N–H group, which makes the molecule resistant to nucleotidyl transfer and hydrolysis. The use of dNMPNPP enables a structural determination of the pre-chemistry state of the polymerase ternary complex with the catalytic metal ion coordinated to the primer terminus 3′-OH. The active site coordination of dNMPNPP is indistinguishable from that of dNTP and has been used in structural studies of various DNA polymerases.

**Figure 4.**
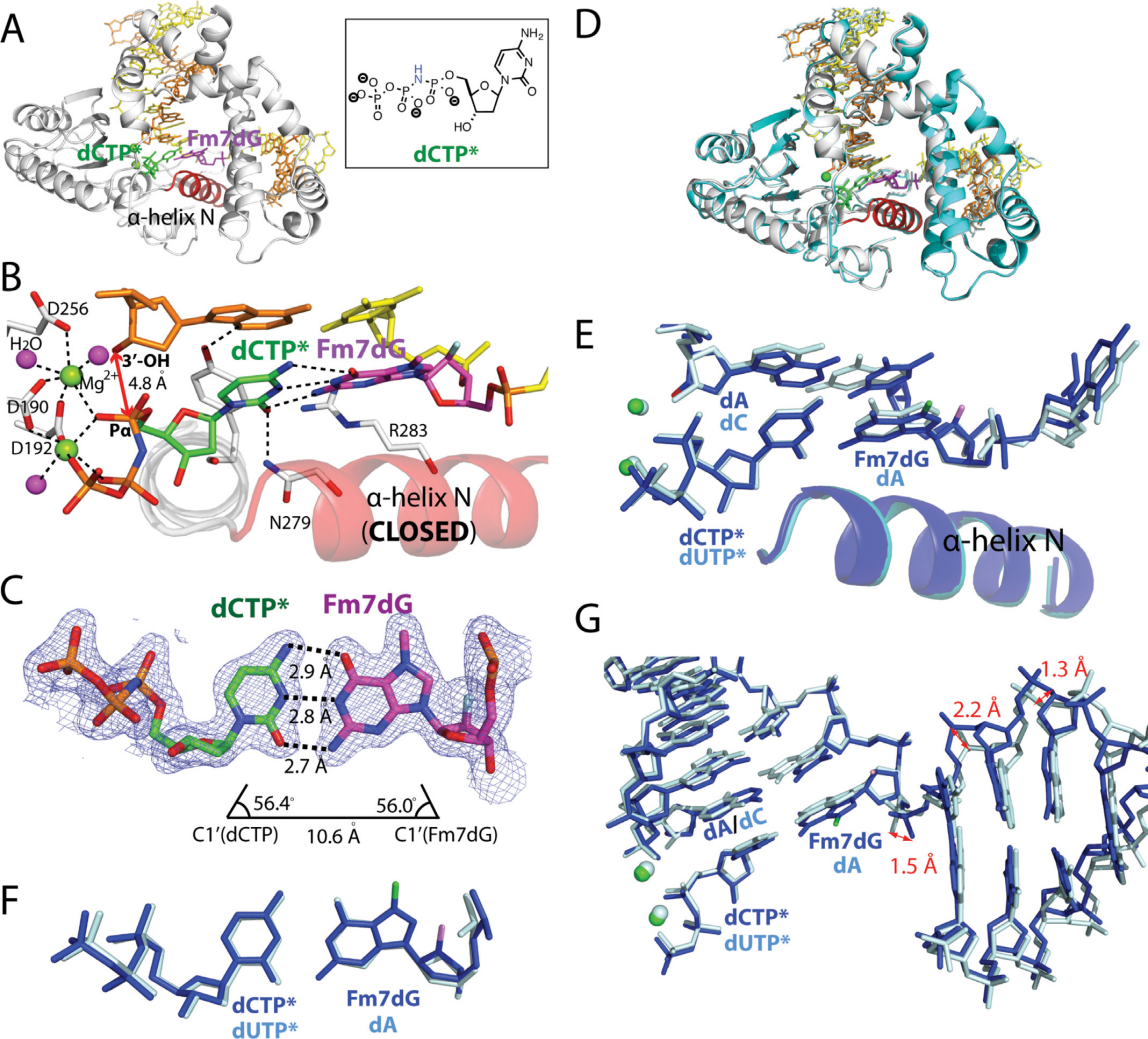
Ternary structure of polβ in complex with templating Fm7dG paired with an incoming nonhydrolyzable dCTP analog (PDB ID: 4O5K). (**A**) Overall structure of the Fm7dG:dCTP* complex. The protein adopts the closed conformation and the nascent base pair adopts a coplanar conformation. (**B**) Close-up view of the active-site structure. The α-helix N is in a closed conformation. Key interactions are indicated as dotted lines. Active-site Mg^2+^ ions are shown as green spheres, and water molecules are shown as magenta spheres. Note that 3′-OH of the primer terminus is not coordinated to the catalytic metal ion. The distance between 3′-OH of the primer terminus and Pα of the incoming nucleotide is indicated. (**C**) Base-pairing mode of Fm7dG:dCTP*. A 2*F*_o_ − *F*_c_ electron density map is contoured at 1*σ* around Fm7dG and dCTP*. The H-bonding distances, the C1(dCTP)′ − C1(Fm7dG)′ distance and *λ* angles are very similar to those observed in a polβ structure with the correct insertion (PDB ID: 2FMS). (**D**) Overlay of the Fm7dG:dTTP*–Mg^2+^ ternary structure with published polβ ternary structure (PDB ID: 2FMS) with nascent dA:dUTP* base pair (RMSD = 0.400 Å) (**E**) Overlay of the active site of the Fm7dG:dTTP*–Mg^2+^ structure (blue) and published dA:dUTP* ternary structure (pale cyan). (**F**) Top view of the Fm7dG:dTTP* base pair (blue) and the dA:dUTP* base pair (pale cyan) in (E). (**G**) Overlay of DNA in the Fm7dG:dTTP*–Mg^2+^ structure (blue) and published dA:dUTP* ternary structure (pale cyan).

The polβ-Fm7dG:dCTP* ternary complex structure, refined to 2.1 Å resolution, shows that Fm7dG:dCTP* base pair is well accommodated in the confines of the polβ active site (Figure 4). The overall structure of the Fm7dG:dCTP* ternary complex (Figure 4A) is essentially identical to that of published ternary structure with correct insertion (PDB ID: 2FMS (25), RMSD = 0.399 Å, Figure 4D). In the Fm7dG:dCTP* complex, polβ undergoes an open-to-closed conformational transition, with α-helix N moving ∼10 Å from the position in the Fm7dG gapped binary complex to sandwich the Fm7dG:dCTP* base pair between the primer terminus base pair and α-helix N (Figure 4B). As observed in published polβ ternary structure with the correct insertion, Tyr271, Asn279 and Arg283 engage in H-bonding interactions with the minor groove edges of the primer terminus base, the incoming nucleotide and the templating base, respectively.

The polβ-Fm7dG:dCTP* structure reveals the base-pairing characteristics of Fm7dG:dCTP* in the confines of the enzyme's active site. Surprisingly, the base-pairing nature of Fm7dG:dCTP* is quite different from that of Fm7dG:dC observed in published AlkA-Fm7dG:dC structure. Unlike a relaxed Watson–Crick Fm7dG:dC base pair with an average H-bond distance of 3.4 Å observed in the AlkA-Fm7dG:dC structure (19), the nascent Fm7dG:dCTP* base pair adopts canonical Watson–Crick conformation with an average H-bond distance of 2.8 Å (Figure 4C). The base pair geometry such as the C1′(Fm7dG)-C1′(dCTP*) distance and the λ angles of Fm7dG:dCTP* is essentially identical to that of canonical Watson–Crick base pair (Figure 4C, E and F). The difference between the base-paring properties of Fm7dG:dCTP* and Fm7dG:dC appears to result from the absence and the presence of constraints by protein. Whereas the Fm7dG:dC base pair in the AlkA-Fm7dG:dC complex is devoid of any interaction with protein, the Fm7dG:dCTP* base pair in the polβ-Fm7dG:dCTP* complex is now in the polβ active site that discriminates between correct and wrong nucleotides using the rigid geometric selection mechanism. The nascent base pair binding pocket with its rigid geometric constraints would suppress the formation of the relaxed base pair conformation observed in the AlkA-Fm7dG:dC structure and promote the formation of canonical Watson–Crick Fm7dG:dCTP* base pair conformation.

Although the Fm7dG:dCTP* ternary complex shares the characteristics of the polβ ternary complex with the correct insertion, the structure indicates that the active-site conformation of this complex is not optimal for nucleotidyl transfer (Figure 4B). Specifically, the distance between Pα of dCTP* and the 3′-OH of the primer terminus is ∼1.4 Å longer than that for correct insertion (25) (4.8 Å versus ∼3.4 Å, Figure 4B). In addition, the primer terminus 3′-OH is not optimally positioned for in-line nucleophilic attack on the Pα of the incoming nucleotide. Furthermore, the critical coordination of the 3′-OH of the primer terminus to the catalytic metal ion (26) is lacking in the Fm7dG:C structure. Instead, an ordered water molecule is liganded to the catalytic metal ion. The coordination of the 3′-OH to the catalytic metal ion has been suggested to lower the p*K*_a_ of the 3′-OH (27) and facilitate proton transfer from the 3′-OH to a catalytic carboxylate residue Asp256 (28). Previous molecular dynamics studies have indicated that nucleophilic attack of the noncoordinated 3′-OH toward the Pα of an incoming nucleotide is energetically highly unfavorable for polβ catalysis (26). The Fm7dG:dCTP* ternary structure thus indicates that, despite its ability to form the canonical Watson–Crick base pairing with the incoming dCTP, the presence of m7dG at the templating position may slow polβ catalysis across the lesion by perturbing the coordination of the catalytic metal ion (Table 1). The lack of coordination of the primer terminus 3′-OH to the catalytic metal ion likely contributes to the observed reduced binding of dCTP opposite the templating Fm7dG (Table 1). The Fm7dG:dCTP* ternary complex will require a subtle conformational adjustment of the active site to reach the catalytically optimal state, and the completion of the coordination sphere of the catalytic metal ion may be a slow or possibly rate-limiting step for polβ catalysis.

**Table 2.** Data collection and refinement statistics

PDB code	Fm7dG binary (4O5C)	Fm7dG:C ternary (4O5K)	Fm7dG:T–Mg^2+^ ternary (4O5E)	Fm7dG:T–Mn^2+^ ternary (4P2H)
Data collection Space group	*P*2_1_	*P*2_1_	*P*2_1_	*P*2_1_
Cell constants				
*a* (Å)	54.396	50.870	54.425	54.767
*b*	79.758	79.568	78.920	79.167
*c*	54.830	55.670	54.819	54.982
*α* (°)	90.00	90.00	90.00	90.00
*β*	105.54	107.71	105.97	106.18
*γ*	90.00	90.00	90.00	90.00
Resolution (Å)^a^	20–2.37	20–2.06	20–2.54	20–1.99
	(2.41–2.37)	(2.10–2.06)	(2.58–2.54)	(2.02–1.99)
<*I*/*σ*>	15.4 (2.66)	15.8 (1.86)	9.7 (1.52)	22.2 (2.52)
Completeness (%)	99.2 (97.1)	98.8 (96.6)	99.7 (97.6)	100 (100)
R_merge_^b^ (%)	10.6 (47.6)	8.2 (52.0)	13.5 (61.1)	8.6 (44.5)
Redundancy	5.5 (5.0)	3.7 (3.5)	3.4 (3.0)	5.6 (5.6)
Refinement				
*R*_work_^c^/*R*_free_^d^ (%)	20.02/24.54	20.03/25.16	20.54/27.28	20.40/24.95
Unique reflections	17819	24650	13821	30969
Mean *B* factor (Å^2^)				
Protein	26.2	23.9	34.7	29.1
Ligand	25.2	32.8	34.8	34.4
Solvent	24.2	28.3	31.5	28.9
Ramachandran plot				
Most favored (%)	97.2	98.5	96.0	97.5
Add. allowed (%)	2.8	1.5	4.0	2.5
RMSD				
Bond lengths (Å)	0.003	0.004	0.005	0.005
Bond angles (º)	0.822	1.124	1.089	1.219

^a^Values in parentheses are for the highest resolution shell.

^b^*R*_merge_ = ∑|*I* − <*I*>|/∑*I*, where *I* is the integrated intensity of a given reflection.

^c^*R*_work_ = ∑|*F*(obs) − *F*(calc)|/∑*F*(obs).

^d^*R*_free_ = ∑|*F*(obs) − *F*(calc)|/∑*F*(obs), calculated using 5% of the data.

How does Fm7dG at the templating position distort the 3′ end of the primer terminus, which is ∼12 Å away from the N7-methyl moiety? The conformation of the incoming dCTP* is nearly indistinguishable from that of an incoming nucleotide in the published structure with the correct insertion (PDB ID: 2FMS (25), Figure 4F), so dCTP* is not likely to cause the perturbation at the primer 3′ end. A comparison of the polβ-Fm7dG:dCTP* complex and the published polβ-dA:dUTP* complex (PDB ID: 2FMS) reveals a significant difference in the conformation of the downstream primer base pairs (Figure 4G), suggesting that the presence of Fm7dG at the templating position induces a considerable conformational change of DNA. Polβ might sense such conformational difference and thus induce a catalytically suboptimal conformation for dCTP incorporation opposite Fm7dG. The perturbation of the 3′ end of the primer terminus has been also observed with published polβ structures with incorrect insertions, showing a longer distance between primer terminus 3′-OH and the catalytic metal ion (e.g. dC:dATP (PDB ID: 3C2L) and dA:dGTP (PDB ID: 3C2M), 4.4 and 4.5 Å, respectively (29)). Mismatched structures also show a staggered base pair conformation and an upstream shift (∼3 Å) of template strand. Overall, our Fm7dG:dCTP* and published mismatched structures illustrate that polβ is highly sensitive to the presence of an abnormality within the enzyme active site and induces an alternate conformation that is suboptimal for chemistry in the presence of active site distortion.

### Ternary structure of polβ incorporating dTTP analog opposite Fm7dG in the presence of Mg^2+^

**Figure 5.**
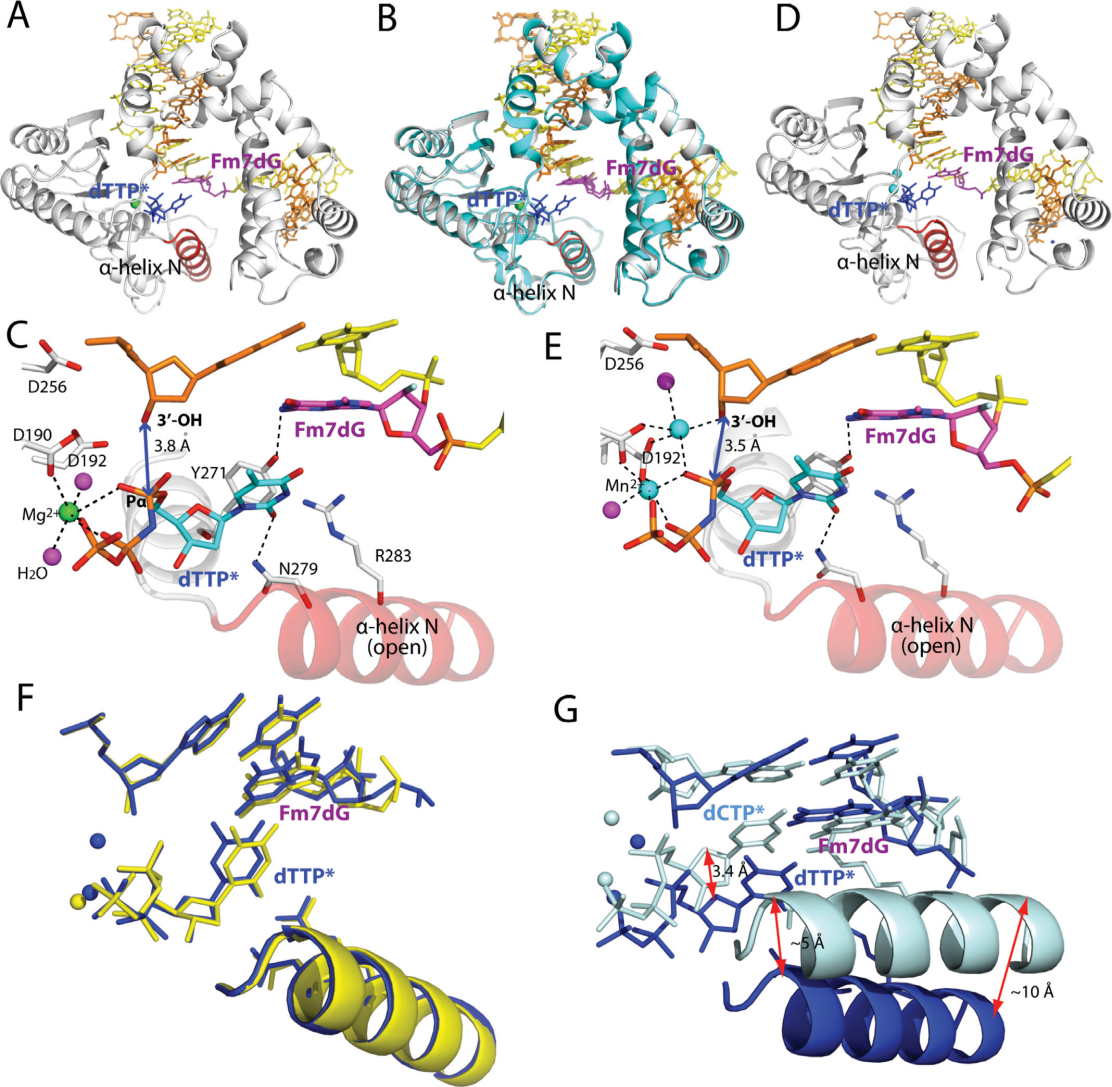
Ternary structure of polβ with templating Fm7dG paired with an incoming nonhydrolyzable dTTP analog (PDB ID: 4O5E and 4P2H). (**A**) Overall structure of the Fm7dG:dTTP*–Mg^2+^ complex. The protein adopts an open conformation, and the nascent base pair adpots a staggered conformation. (**B**) Overlay of the Fm7dG:dTTP*–Mg^2+^ structure (white) with the Fm7dG gapped binary structure (cyan) (RMSD = 0.316 Å). (**C**) Close-up view of the active-site structure of the Fm7dG:dTTP*–Mg^2+^ complex. Fm7dG does not H-bond with the incoming dTTP*. Only the nucleotide-binding metal ion is observed. (**D**) Overall structure of the Fm7dG:dTTP*–Mn^2+^ complex. (**E**) Active-site structure of the Fm7dG:dTTP*–Mn^2+^ complex. Both the nucleotide-binding and the catalytic metal ions are present. (**F**) Comparison of the active site structure of the Fm7dG:dTTP*–Mn^2+^ complex (blue) with that of the Fm7dG:dTTP*–Mg^2+^ complex (yellow). (**G**) Comparison of the active site structure of the Fm7dG:dTTP*–Mn^2+^ complex (blue) with that of the Fm7dG:dCTP*–Mg^2+^ complex (pale cyan).

Previous studies suggested that m7dG may be a promutagenic lesion that can base pair with dTTP through an ionized form of m7dG, which will lead to the formation of pseudo-Watson–Crick m7dG:dTTP base pair (Figure 1) (12,13). To investigate whether a templating m7dG promotes mutagenic replication by forming pseudo-Watson–Crick base pairing with dTTP in the active site of a DNA polymerase, we determined the X-ray structure of polβ in complex with templating Fm7dG base-paired with incoming nonhydrolyzable dTTP analog dTMPNPP (dTTP* hereafter, Figure 5) in the presence of the active-site Mg^2+^.

The Fm7dG:dTTP*–Mg^2+^ ternary structure, solved to 2.5 Å resolution, is significantly different from the Fm7dG:dCTP*–Mg^2+^ ternary structure (RMSD = 1.367Å). The Fm7dG:dTTP*–Mg^2+^ ternary complex adopts a catalytically incompetent state with an open protein conformation, a staggered base pair, and one active-site metal ion (Figure 5A), which is in contrast to the Fm7dG:dCTP* ternary complex with its closed protein conformation, coplanar base pair and two active-site metal ions. The protein and DNA conformations observed in the Fm7dG:dTTP*–Mg^2+^ ternary structure are essentially identical to those of the Fm7dG gapped binary structure (Figure 5B, RMSD = 0.316 Å), indicating that binding of dTTP opposite templating m7dG does not induce any significant conformational change of protein and DNA. More specifically, in the mismatched Fm7dG:dTTP*–Mg^2+^ structure, polβ does not undergo an open-to-closed conformational activation, and the minor-groove recognition amino acid residues (Tyr271, Asn279 and Arg283) do not move from the positions observed in the Fm7dG gapped binary structure. In addition, the incoming dTTP* neither engages in H-bonding interactions with the templating Fm7dG nor stacks with the primer terminus base. Furthermore, the active-site structure shows the binding only of the nucleotide-binding metal ion (Figure 5C) and the absence of coordination of Asp192 to the nucleotide-binding metal ion, which implies that binding of dTTP opposite m7dG in polβ active site is weak.

The Fm7dG:dTTP*–Mg^2+^ structure, which represents a rare polβ ternary structure with an open protein conformation and a single active-site metal ion, strongly suggests that polβ deters the misincorporation of dTTP opposite m7dG by adpoting an alternate conformation that is incompetent for catalysis. This catalytically unfavorable structure suggests that the Fm7dG:dTTP*–Mg^2+^ complex would have much lower accessibility to the catalytically competent state than the m7dG:dCTP complex and that in the polβ active site the formation of the promutagenic pseudo-Watson–Crick m7dG:T base pair is not favored.

### Ternary structure of polβ incorporating dTTP analog opposite Fm7dG in the presence of Mn^2+^

The Fm7dG:dTTP*–Mg^2+^ ternary structure most likely represents a ground-state structure that is unfavorable for nucleotdiyl transfer chemistry (26). To gain insight into the pre-chemistry state of polβ inserting dTTP opposite the templating m7dG, we determined a ternary structure of polβ-Fm7dG:dTTP* in the presence of active-site Mn^2+^, which is known to increase the misincorporation rate of DNA polymerase and facilitate the formation of a closed polβ conformation during misincorporation. The Fm7dG:dTTP*–Mn^2+^ ternary complex was refined to 2.0 Å resolution.

Surprisingly, the Fm7dG:dTTP*–Mn^2+^ ternary and the Fm7dG:dTTP*–Mg^2+^ ternary complex structurres are very similar, with both forming an open protein conformation and a staggered nascent base pair conformation (Figure 5D, RMSD = 0.274 Å). A notable structural difference is found only in the metal ion-binding site. Unlike the Fm7dG:dTTP*–Mg^2+^ complex with only the nucleotide-binding metal ion, the Fm7dG:dTTP*–Mn^2+^ complex contains both the catalytic and the nucleotide-binding metal ions (Figure 5E). However, the catalytic metal ion is not coordinated to Asp256, which is critical for polβ catalysis (28), but is instead coordinated to a water molecule (Figure 5E).

The structural similarity between the Fm7dG:dTTP*–Mg^2+^/Mn^2+^ complexes indicates that substituting Mn^2+^ for Mg^2+^ does not facilitate an open-to-closed conformational transition of the Fm7dG:dTTP* complex (Figure 5F). The Fm7dG:dTTP*–Mn^2+^ structure adopts an open protein conformation that is observed in the Fm7dG gapped binary structure (RMSD = 0.316 Å), indicating the open-to-closed conformational activation is prevented in the presence of dTTP opposite templating m7dG in the active site of polβ. The drastic structural differences between the Fm7dG:dTTP*–Mn^2+^ and the Fm7dG:dCTP*–Mg^2+^ complexes (Figure 5G) indicate that polβ strongly discriminates between m7dG:dTTP and m7dG:dCTP in the enzyme active site and that the templating m7dG does not form the promutagenic pseudo-Watson–Crick base pair with dTTP in the nascent base pair binding pocket of polβ. Consistent with the catalytically incompetent conformation observed in the Fm7dG:dTTP*–Mg^2+^/Mn^2+^ complexes, we were not able to detect the incorporation of dTTP opposite Fm7dG by polβ under various polymerization conditions, indicating that the presence of m7dG in DNA is unlikely to promote mutagenic replication by polβ.

## DISCUSSION

### Base-pairing characteristics of alkylated dG in the confines of the polβ active site

**Figure 6.**
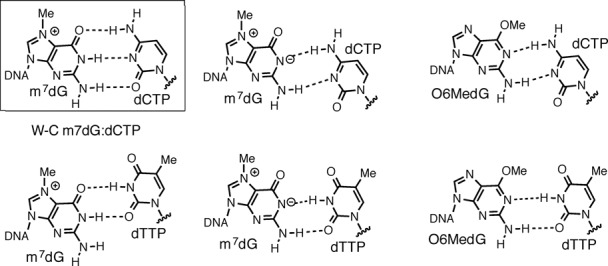
Potential base pairings of a modified dG with an incoming dCTP/dTTP nucleotide. (**A**) Base pairings of m7dG with dCTP/dTTP (**B**) Base pairings of O6-methyl-dG (O6MedG) with dCTP/dTTP. Base pairs observed in the Fm7dG structures and published O6-methyl-dG structures are shown in a box. A polβ structure with the wobble base pair conformation has not been observed.

Our Fm7dG structures and our recently published O6-methyl-dG (O6MedG) structures (30) provide insight into the base-pairing nature of modified dG in the confines of the polβ active site (Figure 6). If the zwitterionic form of Fm7dG exists as a major conformation in the enzyme active site, Fm7dG is likely to facilitate the formation of a wobble base pairing with dCTP* and a pseudo-Watson–Crick base pairing with dTTP* (Figure 6A). However, our Fm7dG structural studies reveal that neither the wobble Fm7dG:dCTP* nor the pseudo-Watson–Crick Fm7dG:dTTP* base pair forms in the enzyme's active site. Instead, the Watson–Crick Fm7dG:dCTP* and the staggered Fm7dG:dTTP* base pair conformations are observed, which indicates that the cationic form of Fm7dG rather than the zwitterionic form of Fm7dG exists as a major isomer in the enzyme active site. It is possible that the electron-rich microenvironment (e.g. π-electrons of nucleobases, phosphate anion) around m7dG may reduce the effect of the N7-methyl moiety on the ionization of N1 of m7dG, thereby suppressing the formation of the zwitterionic m7dG.

Published polβ-O6MedG:dCTP*/dTTP* structures show that the pseudo-Watson–Crick O6MedG:dTTP* base pair, but not the wobble O6MedG:dCTP* base pair, is accommodated in the enzyme active site. Instead, O6MedG:dCTP* forms a staggered base pair conformation (Figure 6B). The failure to observe the wobble base pair conformations of Fm7dG:dCTP*, Fm7dG:dTTP* and O6MedG:dCTP* in the enzyme's active site suggests that polβ strongly discriminates between base pairs with a Watson–Crick-mode geometry and a wobble geometry. Various DNA polymerases such as the A-family DNA polymerase *Bacillus stearothermophilus* DNA polymerase I large fragment (31), the B-family DNA polymerase RB69 (32) and the Y-family DNA polymerase polη (33) have been shown to accommodate wobble G:T or A:C base pair in their active sites. Unlike those polymerases, polβ does not allow the formation of the wobble conformations of A:C, Fm7dG:dCTP/dTTP and O6MedG:dCTP in the active site, highlighting a stringent geometric constraints of polβ. The observation of the pseudo-Watson–Crick base pair conformation for O6MedG:dTTP*, but not for Fm7dG:dTTP* also implies that the zwitterionic form of Fm7dG does not exist as a major conformation in the enzyme's active site.

### Inisight into the replication fidelity mechanism of polβ

**Figure 7.**
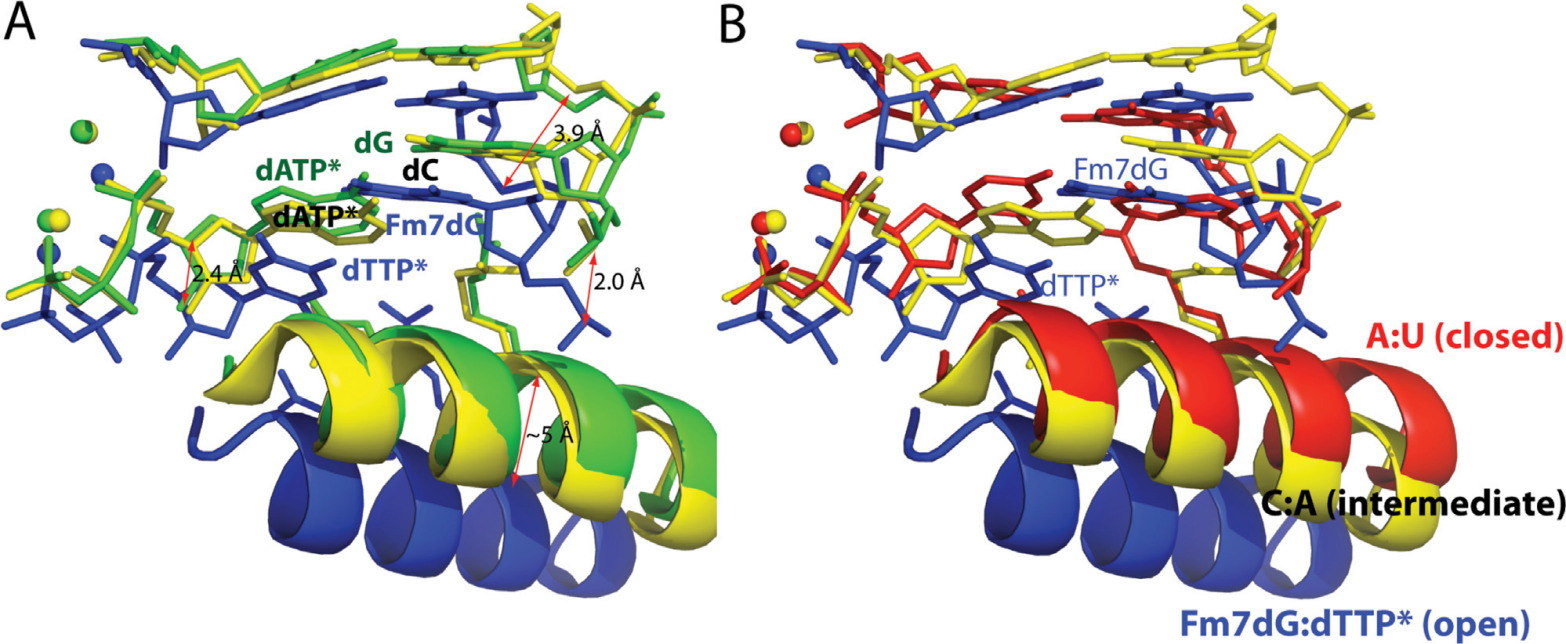
Comparison of the Fm7dG:dTTP*–Mn^2+^ structure with published mismatched structures. Overlay of the Fm7dG:dTTP*–Mn^2+^ structure (blue) with published: (**A**) dC:dATP–Mn^2+^ structure (yellow, PDB ID: 3C2L) and dG:dATP*–Mn^2+^ structure (green, PDB ID: 3C2M), (**B**) dC:dATP*–Mn^2+^ structure (yellow, PDB ID: 3C2L) and dA:dUTP*–Mg^2+^ structure (red, PDB ID: 2FMS).

Comparison of the Fm7dG:dTTP*–Mn^2+^ structure with published polβ structures with base pair mismatch and active-site Mn^2+^ provides insight into the replication fidelity mechanism of polβ (Figure 7). Previous polβ structures with dC:dATP and dG:dATP mismatches show that the enzyme deters nucleotide misincorporation by inducing an alternate conformation that is suboptimal for catalysis (Figure 7A) (22,29). Interestingly, the overall structure of our Fm7dG:dTTP*–Mn^2+^ mismatch complex is very different from those of published polβ ternary complexes with dC:dATP and dG:dATP mismatches (RMSD = 1.610 and 1.515 Å, respectively). Reported mismatched structures show a staggered base pair conformation, an upstream shift (∼3 Å) of template strand near the active site, and a nearly closed protein conformation. Whereas the Fm7dG:dTTP*–Mn^2+^ complex also adopts a staggered base pair conformation, the template DNA strand does not shift significantly, and the protein remains in an open conformation (Figure 7A). In other words, binding of the incoming nucleotide in the enzyme active site induces significant conformational change of protein and DNA for the dC:dATP and the dG:dATP mismatched ternary complexes, but not for the Fm7dG:dTTP* complex. The large conformational differences observed among these structures indicate that the insertion of dTTP opposite m7dG would be less favorable than the insertion of dATP opposite the templating dC or dG. Previous kinetic studies show that dATP incorporation opposite dC and dG by polβ is slow in the presence of active-site Mg^2+^, but increases ∼50-fold in the presence of Mn^2+^ (29). In contrast to those studies, incorporation of dTTP opposite templating Fm7dG is not observed in the presence of either Mg^2+^ or Mn^2+^, which is consistent with the catalytically incompetent conformation observed in the Fm7dG:dTTP*–Mg^2+^/Mn^2+^ structures. The large variation in the active-site conformations of the published mismatched structures and our Fm7dG structures suggests that polβ, which lacks an intrinsic proofreading exonuclease activity, is highly sensitive to the presence of base pair mismatch. It also suggests that polβ increases its replication fidelity by varying the conformations of protein and substrate DNA (Figure 7B). Apparently, polβ deters the formation of the pseudo-Watson–Crick m7dG:dTTP base pair in the enzyme active site while accommodating the Watson–Crick m7dG:dCTP base pair. This indicates that polβ increases its replication fidelity by permitting the closed protein conformation and coplanar base pair conformation only when a nascent base pair can adopt a canonical Watson–Crick base pair conformation.

### Effects of N7-alkyl-dG on DNA replication and mutagenesis

Our kinetic and structural studies, which represent the first systematic investigation of m7dG's mutagenic properties and its effect on polymerase activity, provide insight into the effect of the predominant N7-alkyl-dG on DNA replication and mutagenesis. Recently, Stone *et al.* reported kinetic and structural studies on the Y-family DNA polymerase Dpo4 replicating across the N7-aflatoxin B_1_-dG adduct (34). In the aflatoxin B_1_-dG adduct structure, aflatoxin B_1_ moiety is intrahelical and stacks with the templating dG and an incoming nucleotide, thereby promoting an insertion of dATP opposite the templating N7-aflatoxin B_1_-dG adduct. In addition, the bulky N7-dG acridine half-mustard adduct has been shown to preferentially induce G to A transition mutations (35). As similarly seen in the aflatoxin B_1_-dG adduct structure, the acridine moiety that stacks with an adjacent nucleobase may contribute to the observed transition mutations. Unlike bulky N7-arylalkyl-dG adducts that can form stacking interactions with their aromatic moieties, N7-alkyl-dG lesions with small alkyl groups (e.g. ethyl, propyl) may not be mutagenic, as supported by our kinetic and structural studies on m7dG. The small N7-aklyl-dG lesions may form the canonical Watson–Crick base pair with dCTP in the active site of a DNA polymerase. Our studies also indicate that the presence of small N7-alkyl-dG adducts at the templating position may block replication by some DNA polymerases that are sensitive to an abnormality of the templating base.

### Misincorporation of dTTP opposite m7dG may be less favored than that opposite dG in the polβ active site

Polβ is an error-prone DNA polymerase that makes a mistake every several thousands of the nucleotides transferred. The predominant base substitution errors made by polβ are G:T mismatches, comprising ∼60% of the total base substitution errors (36). The failure to incorporate dTTP opposite the templating Fm7dG by polβ thus implies that the presence of the intact m7dG lesion at the templating position may significantly decrease the dTTP misincorporation rates of some DNA polymerases. The crystal structure of the X-family DNA polymerase polλ, whose active-site structure is essentially identical to that of polβ, shows the formation of a pseudo-Watson–Crick dT:dGTP base pair in the enzyme's active site presumably via ionization (37), implying that a pseudo-Watson–Crick G:T base pair may also form in the polβ active site. Indeed, our polβ ternary structure with dG:dTTP* and active-site Mn^2+^ shows that the templating dG forms a coplanar pseudo-Watson–Crick base pairing with the incoming dTTP* with an average H-bonding distance of 2.9 Å (PDB ID: 4PGX, manuscript in preparation). In addition, our polβ-dG:dTTP*–Mn^2+^ structure shows the closed protein conformation and the completion of coordination of the catalytic metal ion, while the polβ-Fm7dG:dTTP*–Mn^2+^ structure shows an open protein conformation, the incomplete coordination of the catalytic metal ionand the staggered Fm7dG:dTPP* base pair (Figure 5). The large structural differences between the catalytically unfavorable polβ-Fm7dG:dTTP*–Mn^2+^ complex and the catalytically competent polβ-dG:dTTP*–Mn^2+^ complex, together with the lack of dTTP incorporation opposite Fm7dG by polβ, thus suggest that the templating m7dG in the polβ active site deters dTTP misincorporation to a greater extent than the templating dG does.

## ACCESSION NUMBERS

PDB IDs: 4O5C, 4O5K, 4O5E , 4P2H, 1BPX, 1BPY, 2FMS, 3C2L, 3C2M and 4PGX.

## SUPPLEMENTARY DATA

Supplementary Data are available at NAR Online.

SUPPLEMENTARY DATA
