# Supplementary material for: Transition-state destabilization reveals how human DNA polymerase β proceeds across the chemically unstable lesion N7-methylguanine
Source: Nucleic Acids Res. 2014 Jun 25;42(13):8755–66. doi: 10.1093/nar/gku554 (PMC4117778; doi:10.1093/nar/gku554)
Supplement: SUPPLEMENTARY DATA [file supp_42_13_8755__index.html]

Transition-state destabilization reveals how human DNA polymerase β proceeds across the chemically unstable lesion N7-methylguanine — SUPPLEMENTARY DATA 

# Transition-state destabilization reveals how human DNA polymerase β proceeds across the chemically unstable lesion N7-methylguanine

## SUPPLEMENTARY DATA

**Files in this Data Supplement:**

- SUPPLEMENTARY DATA
