# Supplementary material for: Transition-state destabilization reveals how human DNA polymerase β proceeds across the chemically unstable lesion N7-methylguanine
Source: Nucleic Acids Res. 2014 Jun 25;42(13):8755–66. doi: 10.1093/nar/gku554 (PMC4117778; doi:10.1093/nar/gku554)
Supplement: SUPPLEMENTARY DATA [file supp_gku554_nar-01122-h-2014-File003.pdf]

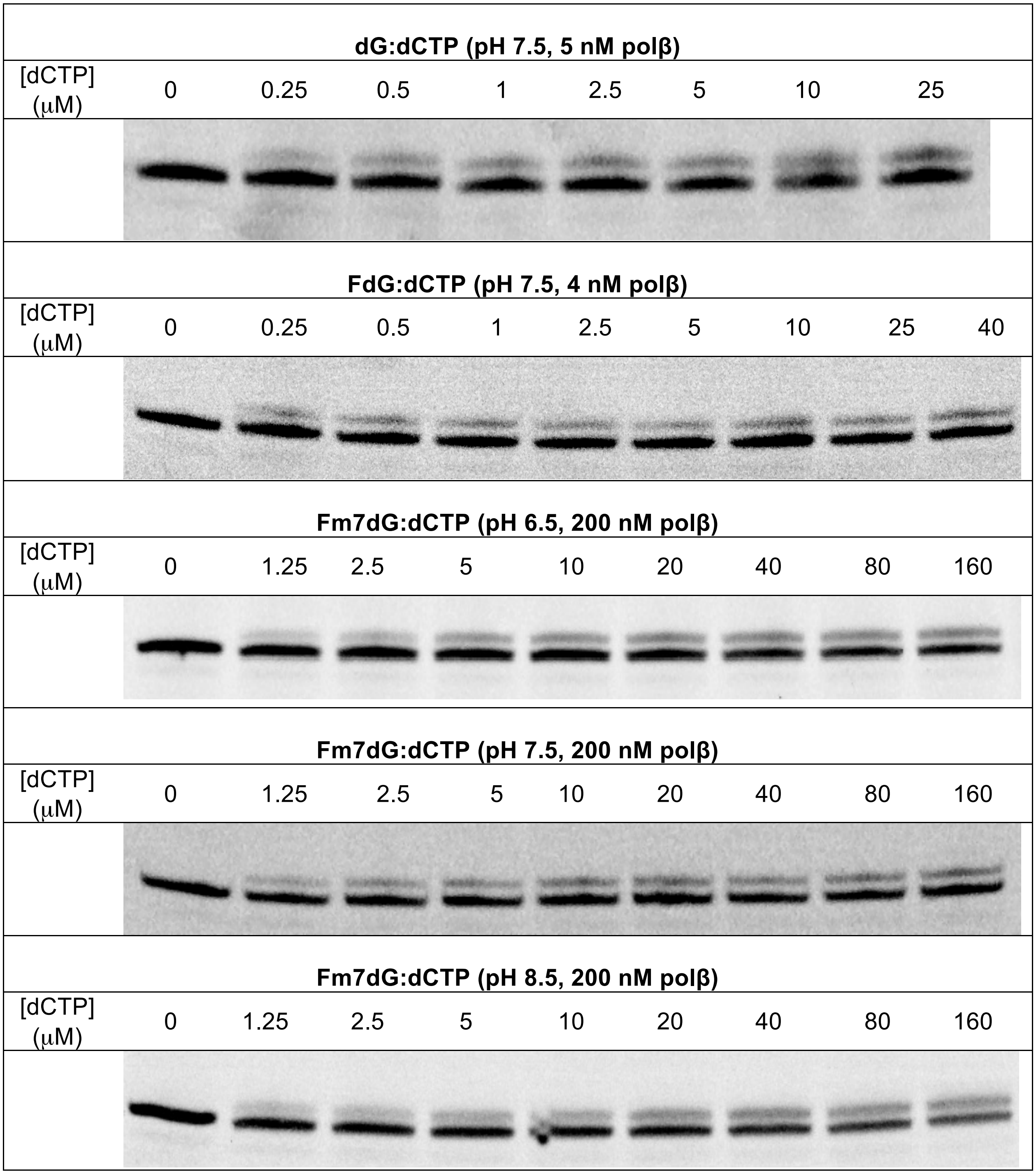

Figure S1. Gel images showing products of single nucleotide incorporation opposite the templating dG, FdG, and Fm7dG by polβ as a function of dCTP concentration. Reactions were initiated by adding dCTP and incubated at 37 °C for 2 min. Reactions were conducted as described in Materials and Methods.
